# Pathologic *RFC1* repeat expansions do not contribute to the development of inflammatory neuropathies

**DOI:** 10.1093/braincomms/fcae163

**Published:** 2024-06-03

**Authors:** Sara Nagy, Aisling Carr, Magdalena Mroczek, Simon Rinaldi, Riccardo Curro, Natalia Dominik, Nicole Japzon, Francesca Magrinelli, Michael P Lunn, Hadi Manji, Mary M Reilly, Andrea Cortese, Henry Houlden

**Affiliations:** Department of Neuromuscular Disease, UCL Queen Square Institute of Neurology, London WC1N 3BG, UK; Department of Neurology, University Hospital Basel, University of Basel, Basel 4031, Switzerland; Department of Neuromuscular Disease, UCL Queen Square Institute of Neurology, London WC1N 3BG, UK; Centre for Neuromuscular Diseases, National Hospital for Neurology and Neurosurgery, Queen Square, London WC1N 3BG, UK; Department of Neurology, University Hospital Basel, University of Basel, Basel 4031, Switzerland; Nuffield Department of Clinical Neurosciences, University of Oxford, Oxford OX3 9DU, UK; Department of Neuromuscular Disease, UCL Queen Square Institute of Neurology, London WC1N 3BG, UK; Department of Neuromuscular Disease, UCL Queen Square Institute of Neurology, London WC1N 3BG, UK; Centre for Neuromuscular Diseases, National Hospital for Neurology and Neurosurgery, Queen Square, London WC1N 3BG, UK; Department of Clinical and Movement Neurosciences, UCL Queen Square Institute of Neurology, University College London, London WC1N 3BG, UK; Department of Neuromuscular Disease, UCL Queen Square Institute of Neurology, London WC1N 3BG, UK; Centre for Neuromuscular Diseases, National Hospital for Neurology and Neurosurgery, Queen Square, London WC1N 3BG, UK; Department of Neuromuscular Disease, UCL Queen Square Institute of Neurology, London WC1N 3BG, UK; Department of Neuromuscular Disease, UCL Queen Square Institute of Neurology, London WC1N 3BG, UK; Centre for Neuromuscular Diseases, National Hospital for Neurology and Neurosurgery, Queen Square, London WC1N 3BG, UK; Department of Neuromuscular Disease, UCL Queen Square Institute of Neurology, London WC1N 3BG, UK; Department of Neuromuscular Disease, UCL Queen Square Institute of Neurology, London WC1N 3BG, UK

**Keywords:** CANVAS, RFC1 disorder, inflammatory neuropathies, chronic inflammatory demyelinating neuropathy, multifocal motor neuropathy

## Abstract

Biallelic expansions of the AAGGG repeat in the replication factor C subunit 1 (*RFC1*) have recently been described to be responsible for cerebellar ataxia, peripheral neuropathy and vestibular areflexia syndrome. This genetic alteration has also allowed genetic classification in up to one-third of cases with idiopathic sensory neuropathy. Here, we screened a well-characterized cohort of inflammatory neuropathy patients for *RFC1* repeat expansions to explore whether RFC1 was increased from background rates and possibly involved in the pathogenesis of inflammatory neuropathy. A total of 259 individuals with inflammatory neuropathy and 243 healthy controls were screened for the AAGGG repeat expansion using short-range flanking PCR and repeat-primed PCR. Cases without amplifiable PCR product on flanking PCR and positive repeat-primed PCR were also tested for the mostly non-pathogenic expansions of the AAAGG and AAAAG repeat units. None of the patients showed biallelic AAGGG expansion of *RFC1*, and their carrier frequency for AAGGG was comparable with controls [*n* = 27 (5.2%) and *n* = 23 (4.7%), respectively; *P* > 0.5]. Data suggest that the pathologic expansions of AAGGG repeats do not contribute to the development of inflammatory neuropathies nor lead to misdiagnosed cases. Accordingly, routine genetic screening for *RFC1* repeat expansion is not indicated in this patient population.

## Teaser

Nagy *et al*. investigated the frequency of *RFC1* repeat expansions in inflammatory neuropathies. No cases with cerebellar ataxia, neuropathy and vestibular areflexia syndrome (CANVAS) could be identified in this patient group, suggesting that this genetic alteration do not contribute to the development of inflammatory neuropathies nor lead to misdiagnosed cases.

## Graphical Abstract

**Graphical Abstract:**
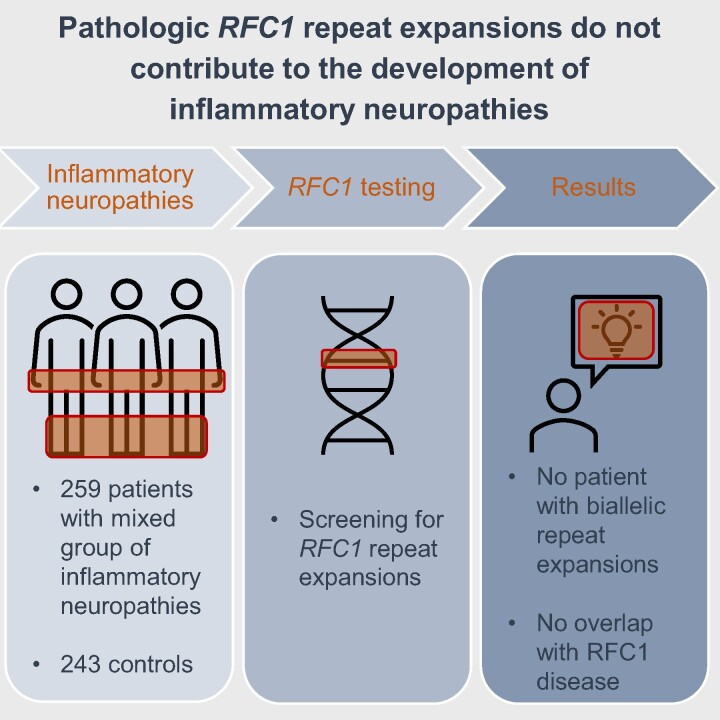

See Siow and Kumar (https://doi.org/10.1093/braincomms/fcae163) for a scientific commentary on this article.

## Introduction

A biallelic pentanucleotide AAGGG repeat expansion in the second intron of the replication factor C subunit 1 gene (*RFC1*) on chromosome 4p14 accounts for CANVAS, a hereditary neurological syndrome characterized by cerebellar ataxia, neuropathy and vestibular areflexia, and, in most cases, chronic cough.^1^ Shorter (<250 repeats) AAGGG repeat expansions as well as AAAAG, AAGAG and AAAGGG repeats are considered as likely normal variants, while there is an expanding number of rarer repeat motifs considered as possibly pathogenic.^2-6^ Clinical data provided evidence that patients with RFC1-related disorders may show a variable clinical manifestation with only two-thirds of CANVAS patients having the full phenotype and 15% of them with isolated sensory neuropathy.^7-9^ Moreover, up to one-third of the genetically unclassified, previously ‘idiopathic’, sensory neuropathy cases have now been classified genetically as CANVAS.^9^ This has led to the screening of more diverse neuropathy cohorts to explore the possibility of undiagnosed CANVAS and expand the phenotype of the *RFC1* repeat expansion genotype. One study described three patients out of 125 with axonal sensory polyneuropathy who carried biallelic *RFC1* expansions and had been misdiagnosed as sensory chronic inflammatory demyelinating neuropathy (CIDP).^9^ Another very recent Japanese study identified four patients having *RFC1* disease out of 240 patients with acute or chronic neuropathies.^10^ However, the late-onset, slowly progressive nature of clinical presentation and neurophysiological findings reflecting dorsal root ganglionopathy, which is the pathological hallmark of this condition, would be at odds with inflammatory aetiologies. Diagnostic criteria for CIDP delineate clinical presentations and neurophysiological features which are differentiating for inflammatory neuropathies, but the application of additional investigations and the documentation of objective evidence of response to treatment are important elements in the diagnosis of these conditions. Clinical evaluation remains the basis of neurology clinical practice. The application of diagnostic tests or other biomarkers must be performed and interpreted in this context.^11^ This becomes increasingly important as we consider the potential pathogenicity of less frequent repeat motifs.^2-6^

Therefore, we aimed to screen a group of well-characterized patients with inflammatory neuropathies according to the diagnostic criteria of the European Academy of Neurology/Peripheral Nerve Society (EAN/PNS)^12,13^ to address whether *RFC1* repeat expansions can (i) contribute to their development or (ii) mimic them, causing diagnostic difficulties.

## Materials and methods

Patients were identified through peripheral nerve sub-specialty consultant neurology inpatient and outpatient services in the UK. A total of 259 patients were included with the following diagnoses: acute inflammatory demyelinating neuropathy (AIDP), CIDP, chronic immune sensory polyradiculopathy (CISP) with normal nerve conduction studies (NCS), multifocal motor neuropathy (MMN) with or without conduction block, autoimmune nodopathy (AN), and combined central and peripheral demyelination (CCPD). For the diagnosis of CIDP, the latest EAN/PNS criteria were considered^12^; therefore, patients with AN and CISP were not regarded as CIDP variants, and those having antibodies against myelin-associated glycoprotein (anti-MAG) were excluded. Demographic, clinical, neurophysiological and follow-up data were collected retrospectively from electronic healthcare records and patient notes. In the control group, 243 healthy UK controls without neuropathy were included.

DNA extraction was performed either from blood or saliva. All samples were tested by flanking polymerase chain reaction (PCR) and repeat-primed PCR (RP-PCR) for AAGGG expansion using the primers, as previously described by Cortese *et al.*^1^ Those samples without an amplifiable product on flanking PCR and positive AAGGG RP-PCR were further tested for the likely non-pathogenic AAAAG and mostly non-pathogenic AAAGG configurations. In addition, a representative number of CIDP cases and controls (*n* = 94) were systematically screened for AAAGG, AAAAG and ACGGG and the recently described pathogenic ACAGG, AAGGC and AGAGG configurations. Statistical analysis of *RFC1* allele frequencies between subgroups was done using Fisher’s exact test.

The study that included the current analysis received approval by the HRA and Health and Care Research Wales. Written informed consent was obtained from all patients. The study was conducted according to the Declaration of Helsinki.

## Results

Adult UK patients were tested (born between 1920 and 2003), of whom 84 (32%) were females and 176 (68%) were males. Age without date of birth was provided in 40 patients; sex was missing in one patient.

The largest group of patients had typical CIDP (*n* = 125), while 10 patients had a CIDP variant [multifocal (*n* = 5), sensory (*n* = 3) or motor (*n* = 2) predominant]. Based on available data, nerve conduction studies were performed in all, and CSF was examined in 62% of CIDP patients. In 19%, a nerve biopsy was obtained which showed no significant alternative pathology and, in most cases, was positively supportive of demyelination and/or inflammation. Seventy-four per cent of CIDP patients had a good response to first-line therapy with immunoglobulins or plasma exchange, while 16% remained unresponsive (10% insufficient data available). Other long-term steroid sparing treatments were given in 28% of patients: in 21% one, in 5% two, and in 2% three or more additional therapies were needed.

**Figure 1:**
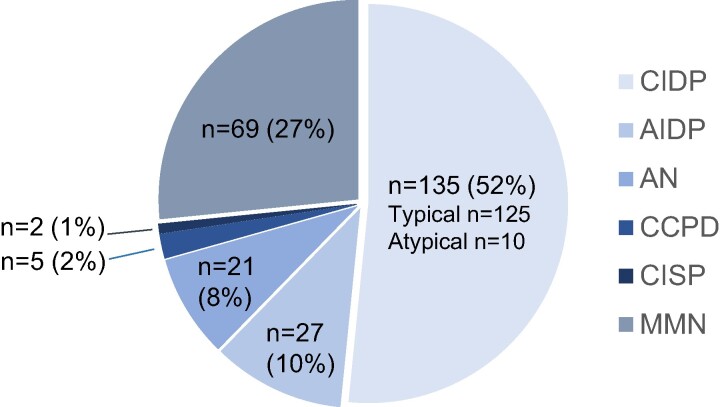
**Inflammatory neuropathy subgroups with patient numbers included in the study**. AIDP, acute inflammatory demyelinating neuropathy; AN, autoimmune nodopathy; CIDP, chronic inflammatory demyelinating neuropathy; CCPD, combined central and peripheral demyelination; CISP, chronic immune sensory polyradiculopathy; MMN, multifocal motor neuropathy.

According to the current criteria,^12^ further, 21 patients were classified with AN (7 patients with anti-neurofascin 155, 1 with anti-neurofascin 140/186, 5 with pan-neurofascin, 6 with anti-contactin-1 and 2 with anti-Caspr1 antibodies). We were also able to identify two patients with CISP and five with CCPD. In the analysis, patients with CIDP variants, AN, CCPD and CISP were grouped together with typical CIDP cases: CIDP+ (*n* = 163). An additional of 69 patients with MMN and 27 with AIDP were included (Fig. 1).

When tested for AAGGG repeats in *RFC1*, none of the patients or controls showed biallelic AAGGG expansions. In total, 27 patients with inflammatory neuropathies were heterozygous for the AAGGG repeat: in the merged CIDP+ group 15 patients, in the MMN group 10 patients and in the AIDP group 2 patients with an allele frequency of 4.8%, 7.2% and 3.7%, respectively (Table 1). Out of the reported 15 CIDP+ cases, 11 had typical CIDP, 2 had a CIDP variant (one multifocal, twot sensory predominant), and further 2 had AN. In the control group, 23 patients were heterozygous for AAGGG with a carrier frequency of 4.7%. No significant difference between the neuropathy groups and the controls could be detected regarding the carrier frequency for AAGGG. Out of those AAGGG carriers with inflammatory neuropathies who had no amplifiable product on flanking PCR, only one tested negative for AAAAG and positive for AAAGG. This patient met all criteria for typical CIDP and did not have any clinical features supporting CANVAS. No significant difference could be seen between CIDP patients and controls regarding the allele frequency of AAAGG and AAAAG repeats (data not shown).

**Table 1:** Allele frequency of the AAGGG repeat expansion in patients with inflammatory neuropathies and controls

	CIDP+ (*n* = 163)	MMN (*n* = 69)	AIDP (*n* = 27)	All (*n* = 260)	Controls (*n* = 243)
AAGGGn ‘heterozygous’	15	10	2	27	23
AAGGGn ‘homozygous’	0	0	0	0	0
Allele frequency (%)	4.6	7.2	3.7	5.2	4.7
*P* value Allele frequency versus controls	>0.99	0.17	>0.99	0.77	NA

Significance level of *P* defined as <0.05.

AIDP, acute inflammatory demyelinating neuropathy; CIDP, chronic inflammatory demyelinating neuropathy; MMN, multifocal motor neuropathy; NA, not applicable.

An additional screening was performed in a representative number (*n* = 94) of typical CIDP cases for the pathogenic ACAGG motif described in the Asian population, as well as for the recently described repeat motifs AGAGG, AAGGC and ACGGG. However, no case tested positive for ACAGG, ACGGG and AAGGC, and only one typical CIDP patient was positive for AGAGG in combination with AAGGG with an amplifiable product on flanking PCR.

## Discussion

The clinical and genetic spectrum of RFC1-related conditions has evolved significantly since its discovery as the genetic basis for CANVAS.^1^ Most patients with biallelic AAGGG expansion exhibit the full CANVAS phenotype of late-onset, slowly progressive cerebellar ataxia, sensory neuropathy, and vestibular areflexia. However, one-third fulfil only one or two of three criteria, also depending on the timepoint and depth of investigations performed.^7,8^ The initially described triad of the classical phenotypic presentation has been expanded to include autonomic dysfunction, pyramidal signs, dystonia, bradykinesia and cognitive impairment in a small proportion of affected patients.^14^ In contrast, sensory neuropathy has been described as *sine qua non* of the disease, with 100% of the patients showing clinical and/or electrophysiological sign of peripheral nerve involvement.^7^

A recent study has explored the frequency of *RFC1* repeat expansions in Japanese patients in the context of inflammatory neuropathies and reported four cases of misdiagnosed CANVAS in this cohort.^10^ However, one patient with the diagnosis of sensory autonomic neuropathy also showed atypical motor involvement, while another patient with slowly progressive anti-MAG neuropathy had only moderate response—partly defined as suppression of IgM levels—to several cycles of immunotherapy.^10^ Some hereditary neuropathies are known to mimic inflammatory neuropathies often enough to be reported. In a large cohort of CIDP patients, 3.2% were diagnosed with CMT (mostly mutations in *PMP22*, *MPZ* and *GJB1*).^15^ Although the coexistence of both conditions cannot be completely excluded, the evaluation of the family history alongside with the pace of disease progression and treatment response is helpful in differentiating. Further, paraclinical findings, including the presence of motor conduction blocks in NCS, contrast enhancement on plexus MRI and high(er) CSF protein levels, are more commonly (but not exclusively) seen in neuropathies of inflammatory origin.^15^ Accordingly, careful phenotyping and application of the EAN/PNS diagnostic criteria for CIDP and GBS helps in identifying inflammatory neuropathies with high sensitivity and specificity.^12,13^ The findings in our study provide further support for their differentiating value.

Here, we present a systematic genetic analysis of *RFC1* repeat expansions in a large, well-characterized cohort on inflammatory neuropathy patients under the care of peripheral nerve sub-specialist consultants in the UK. We found no pathogenic biallelic repeat expansions and a similar frequency of heterozygosity for *RFC1* repeat expansions in inflammatory neuropathy patients and controls. A similar allele frequency (up to 6.8%) has been reported previously in the general population of European origin.^16,17^ Our data suggest that pathologic expansions of AAGGG or any other likely pathogenic repeats are not risk factors for these neuropathies. Moreover, the *RFC1* disease is not commonly misdiagnosed as inflammatory neuropathy.

Nevertheless, diagnosis making in inflammatory neuropathies remains a diagnostic challenge in some situations,^18^ and the coincidental existence of both RFC1 disease and inflammatory neuropathy in patients is certainly possible. However, further genotyping should be limited to cases with atypical clinical presentation, evolution of more CANVAS-like phenotype over time and/or poor treatment response. A similar approach is recommended for patients with CANVAS who have occasionally been misdiagnosed with Sjögren’s sensory ganglionopathy.^9,18^ Later disease onset with a symmetrical and more severe involvement, chronic cough, cerebellar and/or vestibular symptoms and cerebellar atrophy on brain imaging would point towards *RFC1* disease.^19^ Accordingly, the presence of these features would justify genetic testing in those with suspected Sjögren’s syndrome, with the aim of avoiding unnecessary immunotherapy and potential harm to the patient.

Limitations of the study include a retrospective data collection and incomplete data collected on the neurophysiology and objective evidence of treatment response; therefore, current EAN/PNS guidelines could not always be applied. Also, patients were not systematically tested for paranodal antibodies, but considered in the appropriate clinical setting. The frequency of patients having AN reflects previously reported numbers in UK cohorts.^20^

Considering the genotypic spectrum, recent data support the hypothesis that besides biallelic AAGGG expansions, novel repeat motifs including ACAGG and AAGGC expansions in the homozygous state, as well as AAGGG/AAAGG, AAGGG/AGAGG and AAGGG/AGGGGC expansions in the compound heterozygous state, can lead to a CANVAS phenotype.^2-6^ Also, patients with heterozygous AAGGG expansion in combination with a second truncating variant have been described.^21^ In order to interpret the potential pathogenicity of these findings, confidence in the relevance of the phenotype is essential. Although the CANVAS phenotype has expanded, our results support the absence of overlap between inflammatory neuropathies and *RFC1* disease.

## Data Availability

Data may be shared with any qualified investigator upon request.
